# Effective Adsorption of Methyl Orange from Aqueous Solution Using MOFs Nanocomposites UiO-66-NH_2_/GO@PVA

**DOI:** 10.1021/acsomega.5c04958

**Published:** 2025-08-28

**Authors:** Vu Van Thang, Nguyen Tran Duy Nguyen, Phi Long Nguyen, Thi Viet Bac Phung

**Affiliations:** † Center for Environmental Intelligence and College of Engineering and Computer Science, Vin University, Hanoi 100000, Vietnam; ‡ Department of Chemistry, 35016 Soongsil University, Seoul 06978, South Korea; § Hanoi University of Industry, Hanoi 100000, Vietnam

## Abstract

The persistent contamination of water resources by synthetic dyes poses a critical global environmental challenge, necessitating the development of efficient and sustainable remediation technologies. This study presents a novel UiO-66-NH_2_/GO/PVA composite for the effective removal of methyl orange (MO) from water solutions. The composite was created by combining graphene oxide (GO) and UiO-66-NH_2_ in a poly­(vinyl alcohol) (PVA) matrix, creating a stable and very porous structure. Adsorption studies, fitted to the Langmuir isotherm model (*R* ^2^ = 0.9439), revealed a maximum capacity of 188.63 mg/g, with an optimal removal efficiency of 99.6% achieved at an initial MO concentration of 100 mg/L and pH 5. Kinetic analysis confirmed a pseudo-second-order mechanism (*R* ^2^ = 0.9930), indicating chemisorption as the dominant process, while thermodynamic data confirmed the adsorption as both endothermic and spontaneous. The material demonstrated robust reusability, retaining over 95% efficiency after four regeneration cycles. These findings position UiO-66-NH_2_/GO@PVA as a promising, eco-friendly adsorbent for wastewater treatment, with future research planned to evaluate its performance in real or simulated wastewater systems, addressing practical applicability.

## Graphical abstract

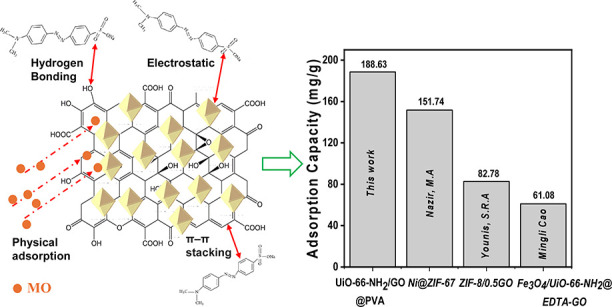

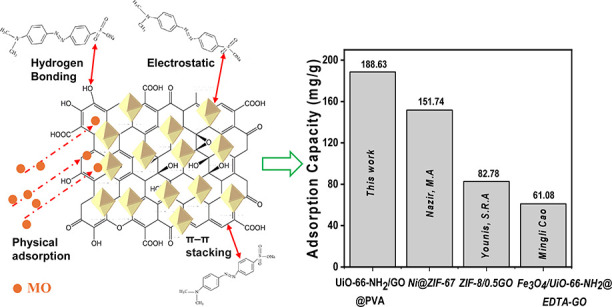

## 1 Introduction

The modern world is becoming more and more industrialized and urbanized, which has resulted in a significant rise in the amount of wastewater that is leaking into the environment containing dangerous contaminants like dyes.^1^ In addition to being visually unappealing, dyes widely employed in the fabric, paper, plastic, and other sectors also pose serious health and environmental hazards.^2^ The dyeing and finishing processes in fabric manufacturing are estimated to contribute 20% of worldwide clean water contamination.^3^ Since many dyes are toxic, carcinogenic, and hard to degrade, eliminating them from wastewater is a serious problem.^4^

Various techniques are now available for removing organic dyes in pollutants, including adsorption,^5^ photocatalysis,^6^ electrocatalysis,^7^ ion exchange,^8^ coagulation,^9^ and membrane filtration.^10^ Adsorption was one of the technologies that was widely utilized since it was relatively inexpensive and highly effective.^11^ In recent years, advanced adsorption technologies have been developed, leveraging novel materials such as 3D metal–organic framework composites (Cu-BTC anchored on MXene nanosheets) for applications like urea adsorption in dialysate regeneration,^12^ ZnO-doped multiwalled carbon nanotubes (MWCNTs) for dye degradation and water purification,^13^ PVDF-based composite membranes for enhanced organic compound separation,^14^ and nanocopper-modified porous carbon for iodine capture.^15^ Among these, Metal–Organic Frameworks (MOFs) have gained attention for dye removal, with UiO-66-NH2 noted for its thermal and chemical stability^16^ due to Zr–O links with dicarboxylate groups^17^ and amine functional groups^18^ (−NH_2_) that enhance adsorption via van der Waals forces, hydrogen bonding, and π–π stacking.^19,20^ This study introduces a novel ternary UiO-66-NH_2_/GO@PVA composite, which, unlike previous binary UiO-66-NH_2_/GO or MOF/PVA systems, synergistically combines GO’s dispersion and adsorption properties with PVA’s mechanical stability, achieving superior adsorption capacity and reusability compared to standalone UiO-66-NH_2_ or binary composites.

However, while the standalone application of UiO-66-NH_2_ is often limited by challenges, such as agglomeration, poor dispersion in aqueous solutions, and limited recyclability, hybrid materials integrating MOFs with other functional components have been explored. The composite MOFs/graphene oxide has potential uses in the wastewater treatment industry.^21^ Graphene oxide (GO) has been recognized as an ideal candidate for improving the performance of MOF-based composites due to its unique 2D structure, huge specific surface area, and richness of oxygen-containing functional groups.^22^ GO not only improves the dispersion of UiO-66-NH_2_ but also contributes additional active sites for adsorption through its functional groups, which can interact with dye molecules via van der Waals forces, π–π stacking, and hydrogen bonding.^23^

In addition to GO, poly­(vinyl alcohol) (PVA) is employed for a polymeric binder in the composite, facilitating the formation of a stable, flexible, and mechanically robust structure.^24^ PVA’s film-forming properties, biocompatibility,^25^ and chemical resistance^26^ enable a porous matrix that supports UiO-66-NH2 and GO integration, enhancing reusability and maintaining high porosity.^27^ While previous studies have combined UiO-66-NH_2_ with GO or PVA individually or in binary composites, our work introduces a ternary UiO-66-NH_2_/GO@PVA nanocomposite with a novel synthesis approach that optimizes the interfacial interactions between components. Specifically, the incorporation of PVA not only enhances the mechanical stability and flexibility but also creates a porous matrix that synergistically amplifies the adsorption sites provided by UiO-66-NH_2_ and GO, distinguishing this composite from earlier binary systems. The uniqueness of this ternary composite lies in its tailored synthesis process, where GO acts as a dispersant and coadsorbent, while PVA serves as a polymeric binder that maintains structural integrity and porosity under repeated adsorption–desorption cycles. This design addresses the limitations of agglomeration and poor recyclability observed in standalone UiO-66-NH_2_ or binary UiO-66-NH_2_/GO composites, offering improved adsorption capacity, enhanced reusability, and greater structural robustness under aqueous conditions.

This work explores the development and use of a MOF composite designed to achieve enhanced wastewater methyl orange (MO) dye removal. UiO-66-NH_2_ and GO are blended throughout the synthesis process, followed by the incorporation of PVA to form a stable and efficient adsorbent. This material offers several advantages, including the exceptional GO ability to adsorb, hydrophilic and flexible PVA, and high porosity and huge surface area of UiO-66-NH_2_. The novel part is how these elements work together to improve the total adsorption capability and effectiveness compared to conventional adsorbents. The composite was examined using XRD, FTIR, SEM, and nitrogen adsorption–desorption to analyze its structure and surface characteristics. Its good and rapid adsorption performance was evaluated through batch experiments on MO dye, examining factors like contact time, dye concentration, and pH. Adsorption kinetics, isotherms, and thermodynamics were analyzed to achieve a deeper understanding of the adsorption behavior. In addition, recyclability and regeneration tests evaluated its practical applicability in wastewater treatment.

In this work, MO was an anionic water-soluble dye that is a significant aromatic pollutant from paper, textiles, and printing industries, among others.^28^ The results of this paper should contribute to the creation of next-generation adsorbents, which offer a viable way to remove dyes from industrial effluents in a sustainable and efficient manner by fusing the stability and adaptability of polymer-based composites with the high efficiency and selection of MOFs.

## 2 Experimental Section

### 2.1 Chemical

Zirconium tetrachloride (ZrCl_4_), poly­(vinyl alcohol) (PVA 99%), methyl orange, and 2-aminobenzene-1,4-dicarboxylic acid (NH_2_–BDC 98%) were supplied by Thermo Fisher Scientific Inc. Graphene oxide (GO), sodium hydroxide, hydrochloric acid, DMF, acetic acid, and ethyl alcohol were purchased from Sinopharm Chemical Reagent Co.

### 2.2 Preparation of UiO66-NH_2_/GO/PVA

UiO-66-NH_2_@GO@PVA was created via modifications to a previously reported hydrothermal method^29^ using a 50 mL Teflon-lined stainless-steel autoclave under a nitrogen atmosphere. Initially, 50 mg of graphene oxide (GO, 99% purity) was dispersed in 20 mL of DMF (99.8% purity) and sonicated at 40 kHz (500 W) for 4 h. The suspension was mixed with 0.64 g of ZrCl_4_ (99.9% purity) and 0.496 g of NH_2_–BDC (98% purity) at a 5 wt % GO ratio, selected based on a previous study that identified 5 wt % as optimal for UiO-66/GO composites,^30,31^ stirred at 300 rpm with a Teflon-coated magnetic stir bar, and adjusted to pH 6.0 with 0.1 M NaOH. The mixture was heated at 393 K for 24 h with a 2 °C/min ramp rate. After cooling, it was washed with 30 mL of ethyl alcohol (99.5% purity) and 30 mL of DMF, centrifuged at 4000 rpm for 10 min, and dried at 393 K for 12 h under nitrogen. Subsequently, 2 g of PVA (99% hydrolyzed, 89,000–98,000 MW) was dissolved in 50 mL of ethanol–water solution (20:80 v/v %, 99.5% ethanol) in a 100 mL round-bottom flask, stirred at 300 rpm for 20 h. The dried UiO-66-NH_2_/GO was added, sonicated at 40 kHz (500 W) for 45 min, and heated at 383 K for 24 h to form the UiO-66-NH2@GO@PVA film. The product was washed with 50 mL of ethanol and 50 mL of deionized water and then air-dried for 24 h.

### 2.3 Equipment and Apparatus

The materials that were synthesized were examined by using a variety of techniques. The structure of PVA@UiO-66-NH_2_/GO was examined using a Hitachi S-4800 FE-SEM scanning electron microscope, transmission electron microscope (TEM) equipped with energy dispersed spectroscopy (EDS) by JEM-2200FS. X-ray diffraction (XRD) spectra of the sample were taken by a D2 Bruker with Cu Kα radiation (λ = 1.5405) to identify the crystalline phase. A Thermo mono model spectrometer was used to get XPS data. Thermo Fisher Nicolet FTIR spectrometers were used to analyze the infrared spectra. The thermal stabilities were ascertained by TGA utilizing the SDT Q600 Auto-DSCQ20 equipment. Approximately 500 mg of the material was dried on a Micromeritics VacPrep 061 degassing machine for 2 h at 130 °C under N_2_. BET (Model TriStar II 3020) was utilized to determine the specific surface area. The zeta potential of the sample was determined using a Zeta potential analyzer SZ-100-Z2. U-VIS spectroscopy (HACH LANGE GmbH DR 2800) was used to study the absorption.

### 2.4 Adsorption Experiments

Adsorption experiments happened at ambient temperature using a shaking device operating at 120 rpm. The pH of the MO solution was set by mixing with 0.01 M HCl or NaOH before starting the adsorption test, and no further adjustments were made. At an initial pH = 5, 5 mg of MOF was introduced into 100 mL of the MO solution (100 mg/L). The starting MO concentration ranged between 10 and 180 mg/L in a 100 mL vial. Kinetic studies and adsorption isotherms were produced when 5 mg of MOFs was introduced to the dye solution at various intervals (5–150 min). After centrifuging the mixture for 5 min at 11,000 rpm, the adsorption was measured using UV/vis spectroscopy. The adsorption models were tested with the help of these data. By conducting adsorption experiments with various initial pH levels ranging from 1.0 to 11.0, we investigated the impact of pH on the ability to adsorb. The ultimate pH was measured when the adsorption experiments were finished. Due to the extremely low ionic strength of the NaOH or HCl injected, the average duplicate data was taken into consideration for determining the impact of pH and performing kinetic testing. Every isotherm experiment was run at least twice.

To evaluate the dye adsorption properties of PVA@UiO-66-NH_2_@GO composites, pseudo-first-order (PFO) and pseudo-second-order (PSO) fits together with Elovich models were presented. The PFO model is represented by the equation that follows [Disp-formula eq1].

$$\ln(q_{e}-q_{t})=\ln q_{e}-k_{1}t$$ 1

According to the PSO model, chemical absorption regulates the adsorption process, with the rate-limiting phase being the electron transfer between an adsorbent and an adsorbate. Eq 2 provides a description of the model’s mathematical formulation.

$$t/q_{t}=1/k_{2}{q_{e}}^{2}+t/q_{e}$$ 2

Here, the adsorption rate constant (*k*_2_) is expressed in terms of g·mg^–1^·min^–1^, and the parameters *q*_e_ and *q_t_* represent the adsorption capacity at equilibrium and at a given time *t* (min).

The Elovich model can be found in eq 3:

$$q_{t}=\frac{1}{b}\ln(a\cdot b\cdot t)$$ 3

where *a* and *b* are the constants about the adsorption rate.^32^

The Langmuir model can be expressed by eq 4:

$$Q_{e}=Q_{m}C_{e}/(1/b+C_{e})$$ 4

The Freundlich model can be expressed by eq 5:

$$Q_{e}=K_{f}{C_{e}}^{N}$$ 5

where *K*_f_ [(mg·g^–1^)/(mg·L^–1^)^*N*^] is the Freundlich isotherm and *N* is the exponential coefficient.^33^

Thermodynamic calculations for dyes adsorption onto MOF composites can be estimated from eqs 6and[Disp-formula eq7]. Δ*G* ^0^, Δ*H* ^0^, and Δ*S* ^0^ are thermodynamic quantities that were found using the van’t Hoff equation.^34^

$$\Delta G^{0}=\Delta H^{0}-T\Delta S^{0}$$ 6

$$\Delta G^{0}=-RT\ln(K_{D})$$ 7

It is possible to use ln *Q*_e_/*C*_e_ to replace the ln *K*_D_ value for the specified temperatures of 298, 313, and 323 K.^35^

## 3 Results and Discussion

### 3.1 Characterization of UiO-66-NH_2_@GO@PVA

The hydrothermal synthesis process of MOF composites is shown in Figure 1. To enhance material stability, PVA was mixed with UiO-66-NH_2_/GO. The MOF composite was introduced as an effective way to adsorb and remove MO.

**Figure 1:**
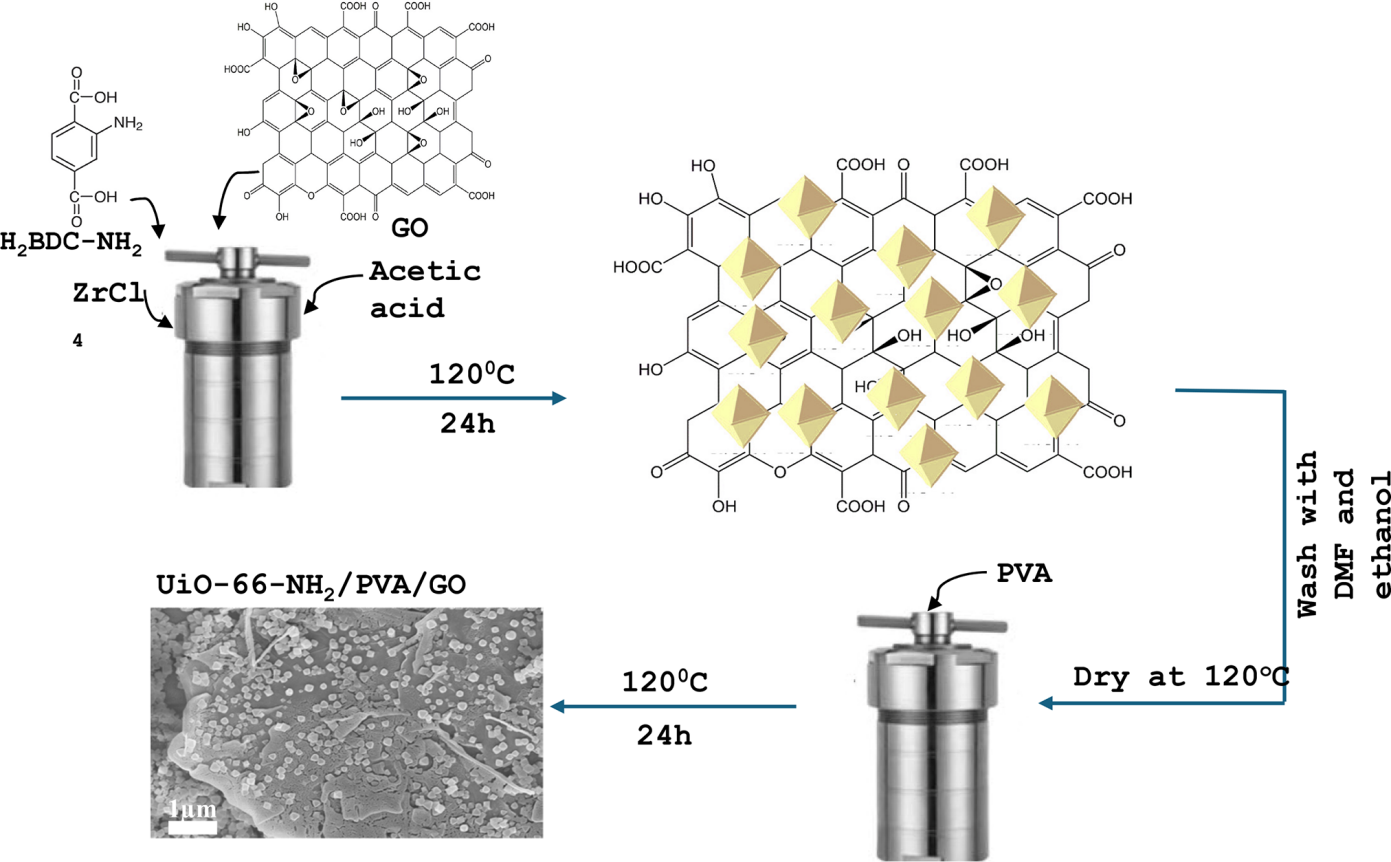
Schematic diagram of the PVA@UiO-66-NH_2_@GO synthesis model.

Morphological analysis of UiO-66-NH_2_/GO/PVA was performed by FE-SEM measurement. UiO-66-NH_2_ has a cubic shape resembling that of UiO-66, which is in line with previous investigations.^36^ UiO-66-NH_2_ particles, approximately 80 nm in size (Figure 2a), were formed by the enclosed space of the GO layers (Figure 2b). This phenomenon occurs because Zr^4+^ fused with the oxygen-containing functional groups in GO, preventing UiO-66-NH_2_ from crystallizing.^37^ Concurrently, the creation of UiO-66-NH_2_ on GO sheets aids in preventing their stacking and makes efficient use of the specific GO structure.^30^ The successful incorporation of PVA into MOFs is demonstrated by the presence of a few PVA fibers in Figure 2c.^38^

**Figure 2:**
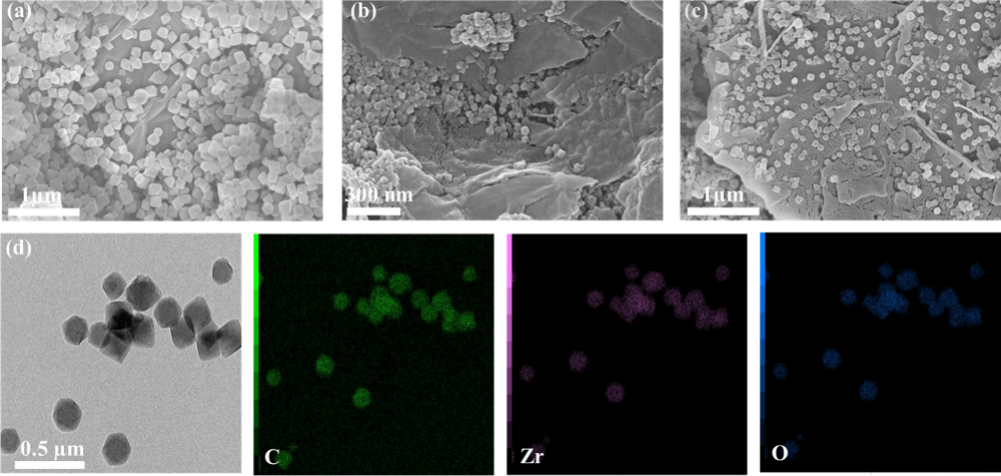
FE-SEM analysis of (a) UiO-66-NH_2_, (b) UiO-66-NH_2_@GO, (c) UiO-66-NH_2_@GO/PVA, and (d) TEM-EDX analysis of UiO-66-NH2@GO/PVA nanocomposites.

TEM-EDX analysis of the UiO-66-NH_2_@GO@PVA composite material revealed its structural and elemental composition (Figure 2d). The TEM image showed uniformly shaped polyhedral nanoparticles with a well-defined morphology confirming their nanoscale size, with an average particle size of approximately 80 nm. The EDX elemental map highlighted the presence of major elements: carbon (C) in green, originating from graphene oxide (GO) and poly­(vinyl alcohol) (PVA); oxygen (O) in blue, associated with oxygen-containing functional groups in GO, PVA, and UiO-66-NH_2_; and zirconium (Zr) in purple, representing the metal centers of the UiO-66-NH_2_ framework. The distribution of these elements confirms the successful incorporation of GO and UiO-66-NH_2_ into the composite material. The relatively uniform dispersion of Zr and C indicates good integration of the metal–organic framework (MOF) and carbon-based components.^29^ The nanoscale size (∼80 nm) enhances adsorption capacity by increasing the surface area and active sites, facilitating efficient interactions with MO via electrostatic attraction and hydrogen bonding, contributing to the observed high adsorption efficiency (99.6%).

FTIR was utilized for qualitative analysis of the functional groups and structure of the synthesized UiO-66-NH_2_/GO@PVA composite, with no peak deconvolution or quantitative intensity comparison performed due to the well-resolved spectra. Figure 3a shows a peak at 3374 cm^–1^ corresponding to the NH_2_ vibration from UiO-66-NH_2_, which facilitates hydrogen bonding with MO’s sulfonate groups and electrostatic interactions with its anionic form.^39^ The peak at 1254 cm^–1^ attributed to the C–N bond supports the MOF framework’s structural integrity, indirectly enhancing adsorption stability.^40^ Bands at 1566 and 1385 cm^–1^ represent asymmetric and symmetric carboxylate vibrations, promoting strong electrostatic interactions with MO’s negative charge, critical for the high adsorption capacity.^41^ Peaks at 480 and 769 cm^–1^ indicate Zr–O bonds, providing robust coordination stability to maintain the composite’s mechanical strength during repeated cycles.^19^ Oxygen-containing functional groups of GO, such as C–O and C=O, likely contribute to π–π stacking and additional hydrogen bonding with MO’s aromatic rings, though their peaks may be suppressed due to interactions with UiO-66-NH2’s metal sites.^42^ The peak at 1159 cm^–1^, corresponding to the C–C vibration from PVA, reinforces the polymer matrix, aiding the uniform dispersion of MOF components and maintaining porosity for efficient MO access. These studies demonstrated the successful chemical synthesis of the MOF composite, with these functional groups collectively driving a high adsorption efficiency of 99.6% via hydrogen bonding, electrostatic interactions, and π–π stacking, as confirmed by FTIR analysis after adsorption in Figure 13b, which shows peak shifts indicative of MO interactions.

**Figure 3:**
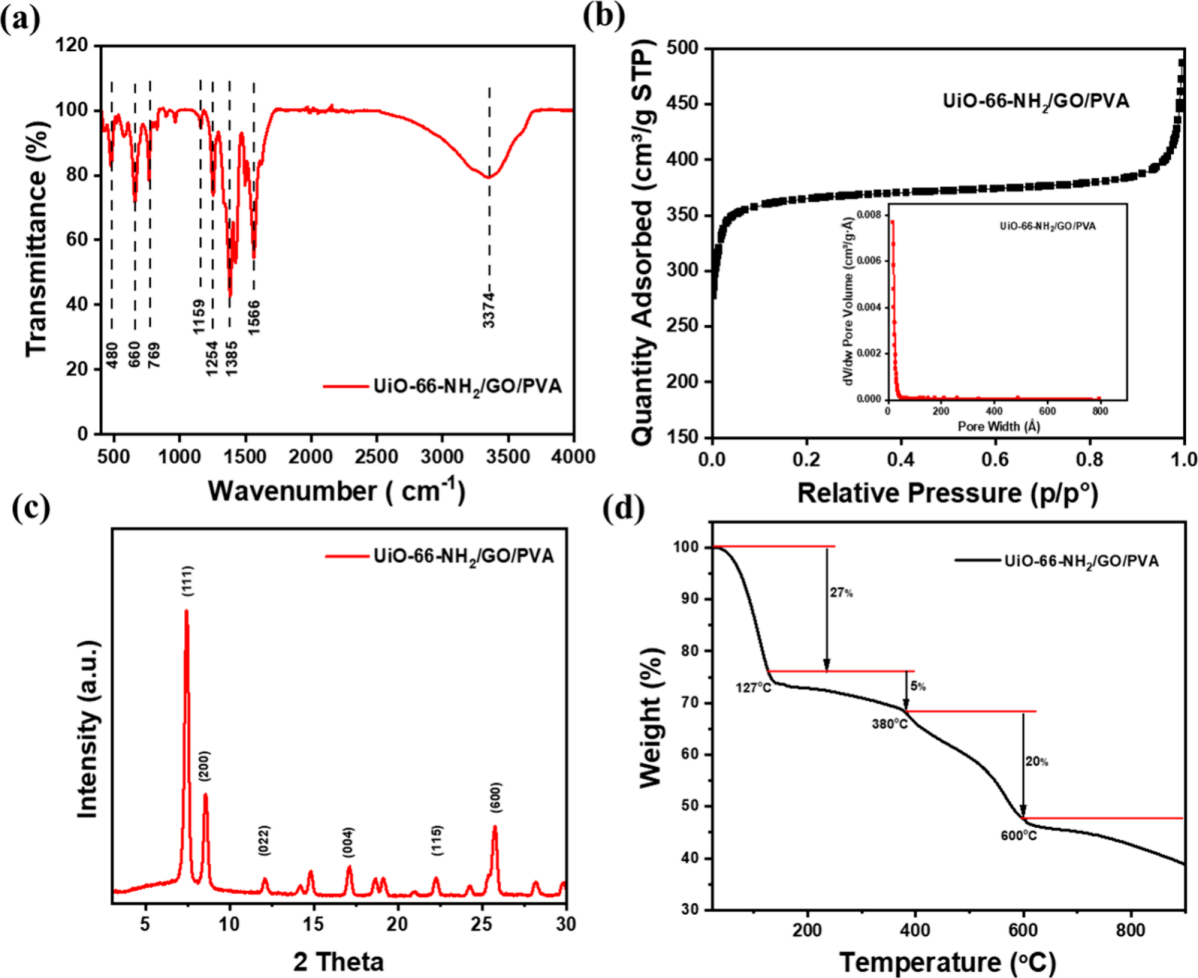
(a) FTIR spectra, (b) BET analysis, (c) XRD spectra, and (d) TGA curves of UiO-66-NH_2_/GO@PVA.

The BET analysis of the sample, shown in Figure 3b, presents a Type I isotherm with a pore size of 2.7 nm showing that the sample has a structure that is extremely porous with microscopic pores.^43^ Additionally, according to the BET statistics, the material MOFs have a surface area of 1129 m^2^/g, and its Langmuir surface area is even greater in 1748 m^2^/g, which denotes the maximum amount of adsorption molecules covered in an even layer on the surface of MOFs. The cumulative pore surface area (118 m^2^/g) and cumulative pore volume (0.22 cm^3^/g) were determined by using the BJH method (Table 1). A single, high-quality measurement was performed due to the instrument’s high reproducibility and the sample’s homogeneity, as validated by consistent XRD, SEM, and FTIR results. No repeated measurements or standard deviation was obtained, as the well-defined isotherm and adherence to standard protocols ensured data reliability. The high surface area is attributed to the synergistic contributions of UiO-66-NH2’s porosity, GO’s large surface area, and PVA’s enhanced dispersion.^30^

**Table 1:** BET Parameters of the Sample

sample	pore size (nm)	*S*_BET_ (m^2^/g)	*S*_Langmuir_ (m^2^/g)	cumulative pore volume (cm^3^/g)	cumulative pore surface area (m^2^/g)
UiO-66-NH_2_/GO@PVA	2.7	1129	1748	0.22	118

Figure 3c shows the XRD spectrum of the MOFs materials; the presence of characteristic peaks at 7.46°, 8.6°, 12.04°, 17.18°, 18.6°, 22.28°, and 25.74° provides evidence of the sample synthesizing.^44^ The TGA analysis was also used to investigate the thermal characteristics of the sample in Figure 3d. In its initial state, which ranged from 23 to 127 °C, the weight of the tested sample dropped by 27% according to the amount of moisture in the sample. The second state, which described a nearly 5% breakdown of physically absorbed DMF in the absorbent, was seen at temperatures between 149 and 380 °C. Subsequently, between 380 and 600 °C, the organic ligand broke down, resulting in a 20% decrease in weight (2-amino-terephthalic acid). The final step induced a deterioration of about 9%.^45^

Using XPS, we identified the surface chemical state of the sample. The MOFs contained C, N, O, and Zr, as shown by the XPS spectra (Figure 4). Two distinct diffraction peaks, Zr 3d_3/2_ and Zr 3d_5/2_, can be seen in the Zr 3d spectra of the MOFs material at 184.38 and 181.98 eV (Figure 7).^46^ The C 1s spectrum is likely to be identified into three peaks: C–C (283.98 eV), C–O (285.38 eV), and C=O (287.98 eV),^47^ while the cross-linking of the PVA substrates is responsible for the C–O peak at 284 eV and the C=O peak at 285.38 eV is associated with the UiO-66-NH_2_/GO doubly linked nanoparticles.^38^ The successful construction of the MOFs material was therefore demonstrated by the findings of the XPS examination.

**Figure 4:**
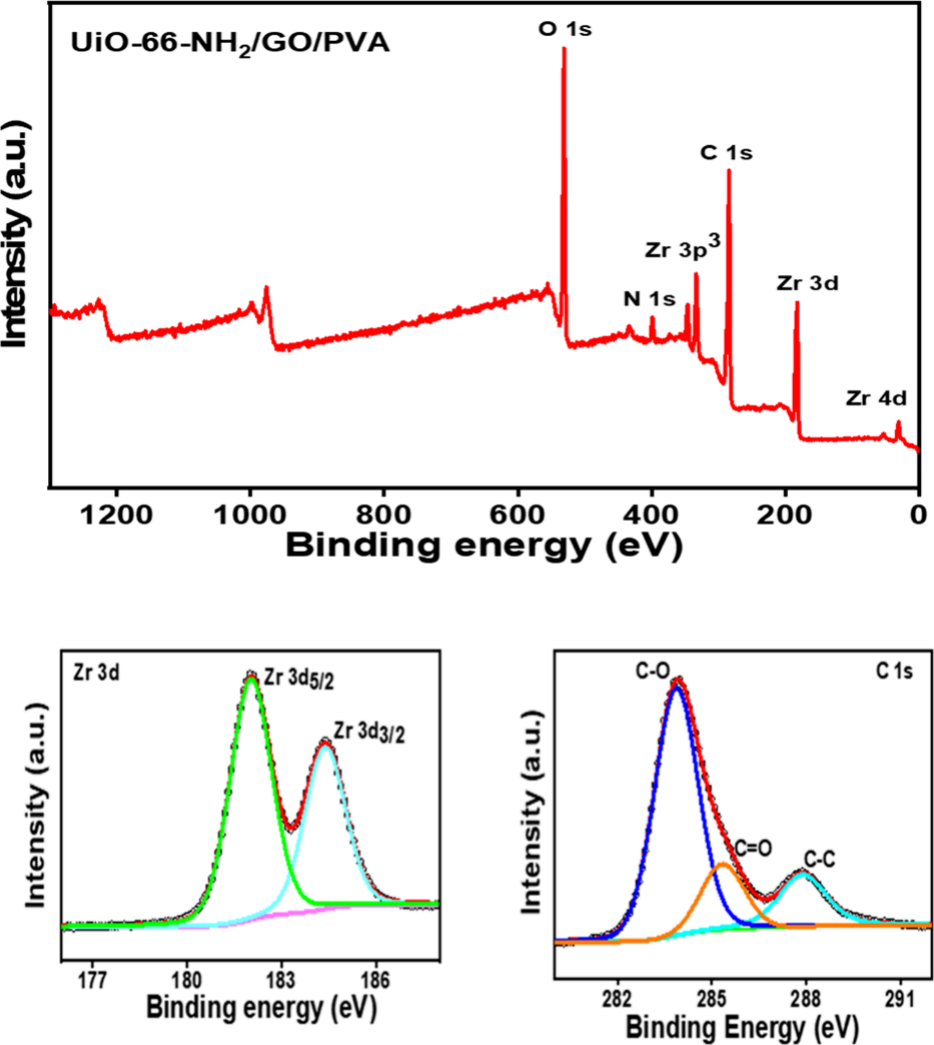
XPS analysis of UiO-66-NH_2_/GO/PVA (Wide scan, Zr 3d, C 1s).

To highlight the novelty and improved performance of the UiO-66-NH_2_/GO@PVA composite, a comparative analysis with recently reported MOF-based hybrid adsorbents is presented in Table 2. This comparison focuses on adsorption capacity, reusability, and structural robustness, which are critical for practical wastewater treatment applications. The UiO-66-NH_2_/GO@PVA composite exhibits superior adsorption capacity and reusability compared to previously reported binary systems, attributed to the synergistic effect of the ternary structure and the stabilizing role of PVA. These enhancements are further validated by the material’s high surface area (1129 m^2^/g) and maintained porosity, as discussed in the characterization section. The following sections evaluate the adsorption performance under various conditions to corroborate these improvements.

**Table 2:** Comparison with Existing MOF-Based Adsorbents

ref	material	adsorption capacity (mg/g)	reusability (cycles, % efficiency)	structural robustness
this study	UiO-66-NH_2_/GO@PVA	188.63	4, >95%	high (stable after 4 cycles)
^48^	Ni@ZIF-67	151.74	5, >90%	good
^49^	PAA–PVA/PW_12_ @UiO-66 NFM	not mentioned	5, >92%	good
^50^	ZIF-8/0.5GO	82.78	not mentioned	good

### 3.2 Study of the Adsorption Parameters

#### 3.2.1 Effect of Dosage on Removal

Figure 5 illustrates the effect of different adsorbent doses on the MO removal under specific test conditions. The effects of the sample dosage were investigated by adjusting the dosage from 0.5 to 12 mg mixed with 100 mL of MO concentration (100 mg L^–1^) at ambient temperature. As the MOFs dosage rises from 0.5 to 5 mg, the removal efficiency significantly and approaches 100%. This implies that more active sites are available for MO absorption at greater adsorbent doses. Above 5 mg, adsorption stabilizes, and efficiency marginally decreases at 12 mg, probably because of agglomeration that reduces the available surface area. For maximal clearance efficacy, the ideal dosage seems to be between 5 and 10 mg.

**Figure 5:**
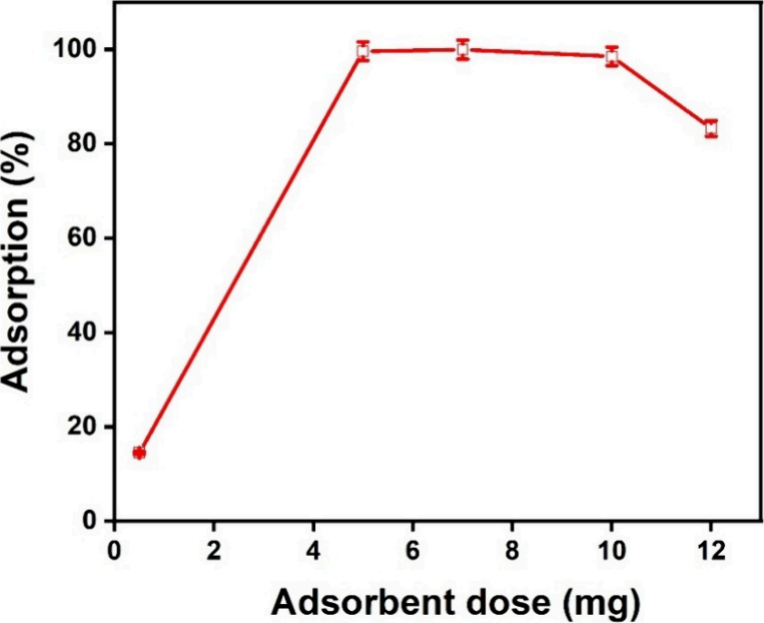
Effect of the dosage on the removal efficiency of MB.

Following equilibrium, the MO elimination efficiency significantly increased from 18 to 99% as the MOFs dosage was raised from 0.5 to 10 mg. At higher adsorbent doses, this improvement is ascribed to the larger surface area and the greater number of accessible adsorption sites. But beyond 10 mg, there was a minor drop in adsorption effectiveness, most likely due to agglomeration of adsorbent particles, which lowers the effective surface area and prevents more adsorption.^51^ The dosage effect was studied by varying adsorbent mass from 0.5 to 12 mg in 100 mL of 100 mg/L MO solution, with removal efficiency measured via UV/vis spectroscopy after centrifugation at 11,000 rpm for 5 min. Units are reported as mg (dosage) and % (efficiency), with duplicates averaged to ensure reliability. Additionally, diffusion and mass transfer limitations may contribute, as the microporous structure (2.7 nm pores, Table 1) restricts intraparticle diffusion, and agglomeration hinders mass transfer from the solution to the surface.^52^

#### 3.2.2 Effect of Contact Time and Initial Concentration

The effect of starting MO concentrations (50, 100, and 160 mg/L) on the UiO-66-NH_2_/GO/PVA ability to adsorb over time is displayed in Figure 6. During the first 20 min, the adsorption capacity (*Q_t_*) rises quickly before equilibrating gradually, and the required equilibrium time is about 120 min. Among the three concentrations at equilibrium, the highest adsorption capability 90.25 is shown by 100 mg/L, followed by 50 mg/L 55.12 and 160 mg/L 43.34. Competitive adsorption effects or surface saturation could be the cause of the reduced adsorption at 160 mg/L.^53^ Contact time (0–150 min) and initial concentrations (50, 100, 160 mg/L) were tested with 5 mg of adsorbent in 100 mL of solution, with *q_t_* (mg/g) calculated from absorbance changes. Equilibrium at 120 min was determined by plateauing *q_t_* values with duplicates averaged. According to this, the material makes good use of its available adsorption sites at an ideal concentration of 100 mg/L, whereas concentrations that are too high may result in decreased effectiveness.

**Figure 6:**
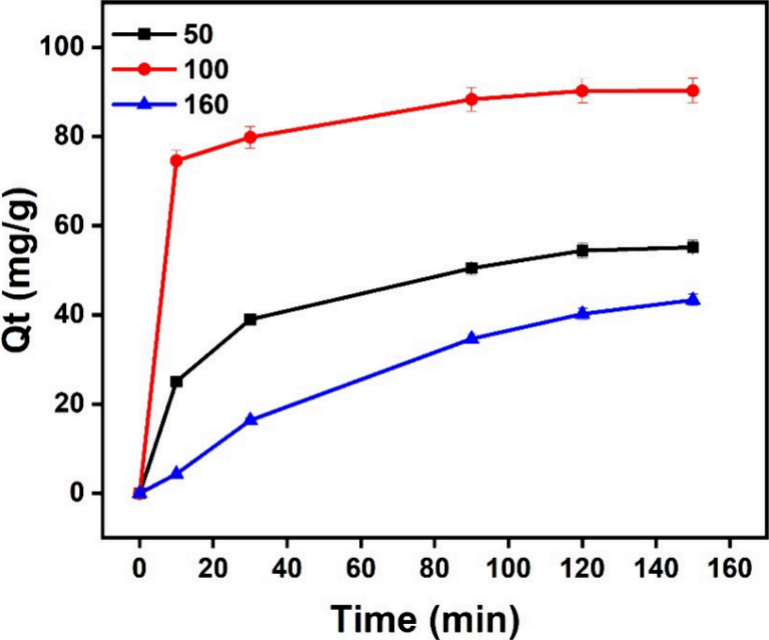
Effect of the contact time and initial concentration on adsorption.

#### 3.2.3 Effect of pH Values

The initial pH of the solution alters the ionization state of MO and the surface charge of the adsorbent, significantly impacting the adsorption performance.^54^ Various pH levels were used to assess the impact while keeping all other factors constant (Figure 7a). At pH = 5, the highest MO adsorption percentage was noted because MO existed in an anionic form at that pH,^55^ which also is in line with the adsorption kinetics and isotherm. When the dye pH was raised from 5 to 11, the adsorption effectiveness of MOFs dropped from 96.32 to 23.02% because of the large repulsion force caused by the OH^–1^ ions of MO. pH was adjusted from 1.0 to 11.0 using 0.01 M HCl or NaOH, with adsorption efficiency (%) measured after 180 min using a 5 mg adsorbent in 100 mL of 100 mg/L MO. Zeta potential (mV) was measured to correlate surface charge with pH, with duplicate data averaged. In summary, the removal of MO adsorption by MOFs materials is facilitated by an acidic pH. The zeta potential of MOFs materials at various pH values is shown in Figure 7b. In acidic conditions, the surface of MOFs appears more positive charges, which favors the MO adsorption of MOFs materials. As the solution pH approaches 5, the zeta potential is at greatest, significantly enhancing the adsorption of MO anions.

**Figure 7:**
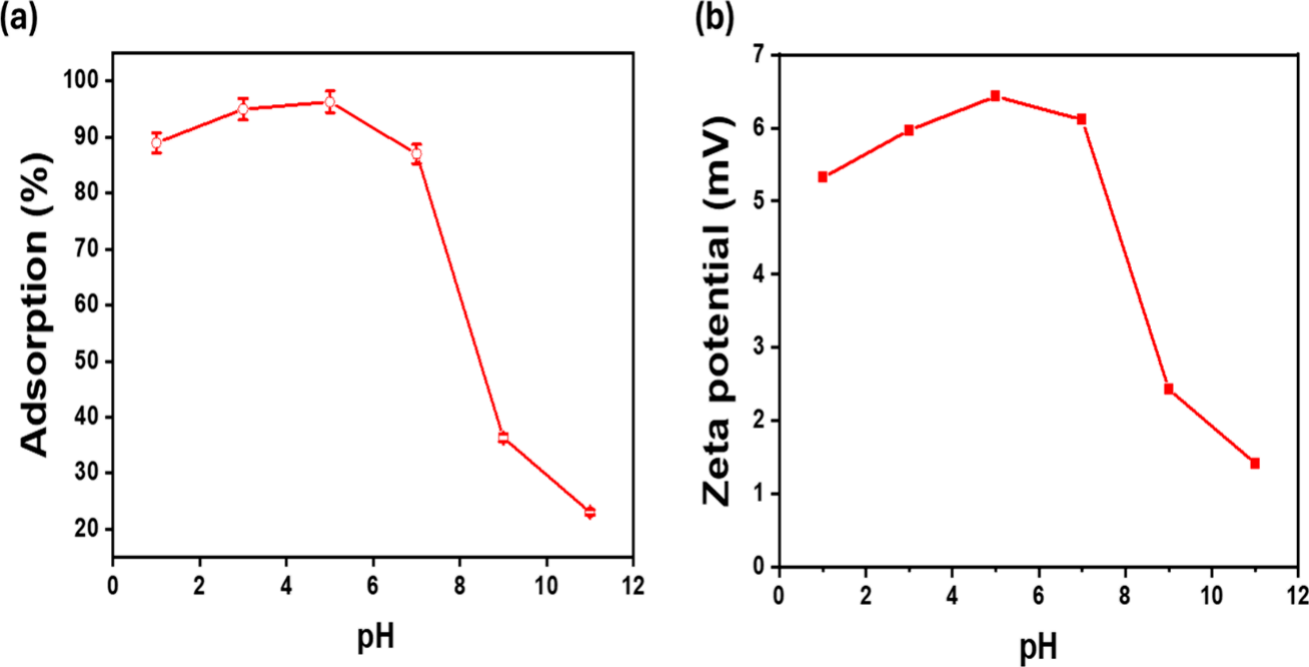
(a) Effect of pH adsorption of MO and (b) Zeta potential of UiO-66-NH_2_/GO/PVA at different pH values.

### 3.3 Adsorption Kinetic, Isotherm, and Thermodynamic Studies

UV/vis spectrophotometry was used to create a standard curve that showed the concentration of MO in the solutions. MO solutions ranging in concentration from 0 to 150 mg/L were prepared, and their absorbance values were measured. The standard curve, depicted in Figure 8, was generated by plotting the absorbance against the corresponding MO concentrations. This calibration curve served as a quantitative tool for estimating the MO concentration in subsequent experiments.

**Figure 8:**
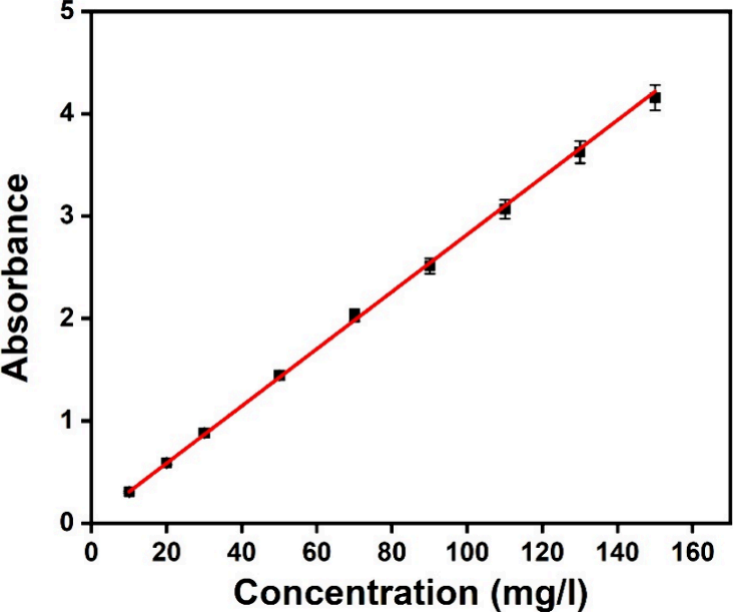
Linear plot of absorbance vs concentration of the MO.

Onto the UiO-66-NH_2_/GO/PVA, MO adsorption exhibited a strong interaction, with adsorption increasing progressively over time and reaching equilibrium at 120 min (Figure 9). To analyze the adsorption kinetics, several models were applied, including the Elovich model, pseudo-first-order (PFO), and pseudo-second-order (PSO) models. The PSO model (assuming physisorption, *q*_*t*_ = 0 at *t* = 0) fits the data of the experiments best, as indicated by a high correlation coefficient (*R* ^2^ = 0.993). MOFs have an initial adsorption rate (*v*_0_) of 0.00421 mg/(g·min) and an equilibrium adsorption capacity (*Q*_e_) of 90.897 mg/g, as determined by the PSO model (assuming chemisorption with electron transfer, *q*_*t*_ → *q*_e_ as *t* → ∞). These findings suggest that chemisorption governs the adsorption process, highlighting the efficiency of the composite material for MO removal.

**Figure 9:**
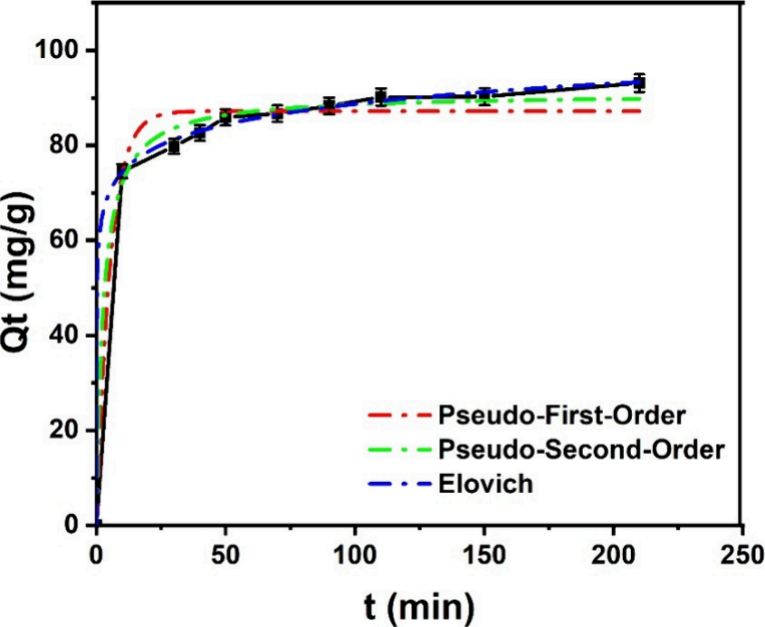
Kinetic adsorption modeling for MO with an initial concentration of 100 mg/L and a contact time of 0–150 min at pH = 5.

The adsorption kinetics of MO onto MOFs composites were thoroughly investigated using the PSO and Elovich models. The Elovich model (assuming heterogeneous adsorption), with the highest correlation coefficient (*R* ^2^ = 0.9999), provides the best fit to the experimental data, indicating a heterogeneous adsorption process governed by activated chemisorption on a diverse surface. This is closely linked to the composite’s characterization: the high surface area (1129 m^2^/g) and microporous structure (2.7 nm pores, Table 1) from BET analysis (Figure 3b) enhance the availability of active sites, while the uniform dispersion of UiO-66-NH_2_ cubic particles and GO layers, reinforced by PVA fibers (SEM, Figure 2), facilitates multisite interactions. The presence of functional groups (−NH_2_ at 3374 cm^–1^, C=O, and Zr–O at 769 cm^–1^ from FTIR, Figure 3a), confirmed by XRD crystallinity (Figure 3c), supports diverse interactions (hydrogen bonding, electrostatic attraction) with MO’s azo and sulfonate groups, as evidenced by FTIR shifts postadsorption (Figure 13b). The PSO model (*R* ^2^ = 0.9930), while offering a strong fit, is less representative, suggesting that electron transfer-based chemisorption is a secondary contribution, providing additional evidence of chemical interactions between MO and MOFs.^56^ The Elovich model, in conjunction with the Freundlich and Langmuir isotherm models, characterized adsorption as an activated chemisorption mechanism. This comprehensive analysis, including kinetic and isotherm studies, offers a detailed understanding of the MOFs adsorption behavior. Table 3 summarizes the key kinetic parameters and adsorption characteristics.

**Table 3:** Kinetic Models Parameters

models	parameters	values
PFO	*Q*_e_ (mg/g)	87.200
	*k*_1_ (min^–1^)	0.1887
	*R* ^2^	0.9791
PSO	*Q*_e_ (mg/g)	90.897
	*v*_0_ (mg/(g**·**min))	0.00421
	*R* ^2^	0.9930
Elovich	*a*	94793.232
	*b* (g/mg)	0.165
	*R* ^2^	0.9999

Figure 10 illustrates the modeled isotherms depicting MO adsorption onto UiO-66-NH_2_/GO/PVA. According to the Langmuir model (assuming monolayer coverage on uniform sites, *C*_e_ = 0 at no adsorption), the maximal theoretical adsorption ability of the sample is 188.63 mg/g. Additionally, the Freundlich isotherm (assuming multilayer adsorption on heterogeneous sites) parameter (*N*) suggests surface heterogeneity, with lower values indicating a more heterogeneous surface. The fitted Freundlich isotherm for MOFs shows an *N* value of 3.05, confirming the presence of diverse adsorption sites (Table 4). This result further indicates that the incorporation of MO as a secondary linker alters the surface characteristics. Overall, these results show the modifications to the structure induced by the addition of PVA to improve its capacity to remove MO by adsorption.

**Figure 10:**
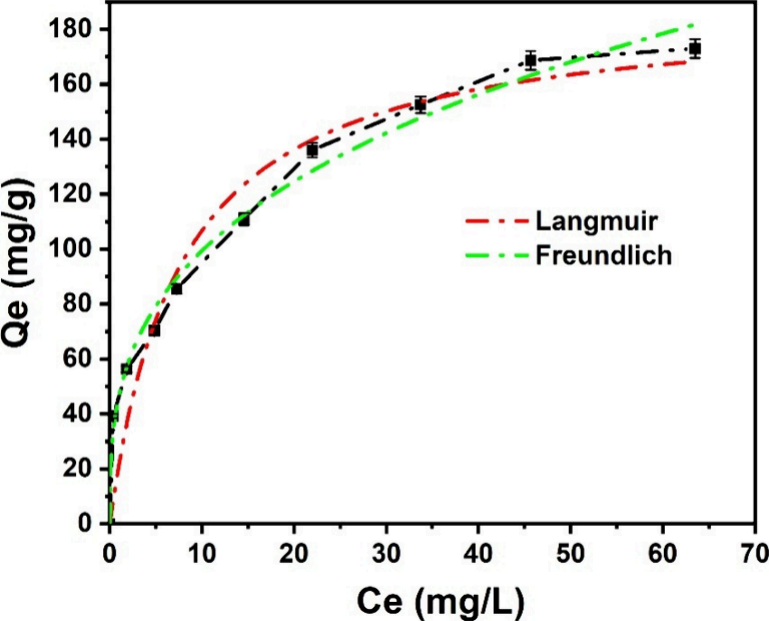
Adsorption isotherm of MO onto UiO-66-NH_2_/GO/PVA. Experimental conditions: contact time: 180 min, adsorbent mass: 5 mg, equilibrium concentration 0–150 mg/L, and pH= 5.

**Table 4:** Isotherm Models Parameters

models	parameters	values
Langmuir	*Q*_m_ (mg/g)	188.6313
	*b* (L/mg)	0.1302
	*R* ^2^	0.9439
Freundlich	*K*_f_ ((mg/g)/(mg/L)^*N*^)	46.8169
	*N*	3.0599
	*R* ^2^	0.9891

An overview of the determined thermodynamic parameters is shown in Table 5 and Figure 11. A negative Δ*G* ^0^ value suggests that both MOFs and MO interactions are spontaneous.^57^ The magnitude of Δ*G* ^0^ also helps differentiate between physisorption and chemisorption. In this study, Δ*G* ^0^ values ranged from −40 to 0 kJ/mol, confirming that the adsorption process for the UiO-66-NH_2_/GO/PVA adsorbent followed physisorption.^58^

**Table 5:** Thermodynamic Parameters

*T* (K)	Δ*G* ^0^ (kJ/mol)	Δ*H* ^0^ (kJ/mol)	Δ*S* ^0^ (kJ/mol·K)
298	–4.44	32.14	112.30
313	–6.02		
323	–7.27		

**Figure 11:**
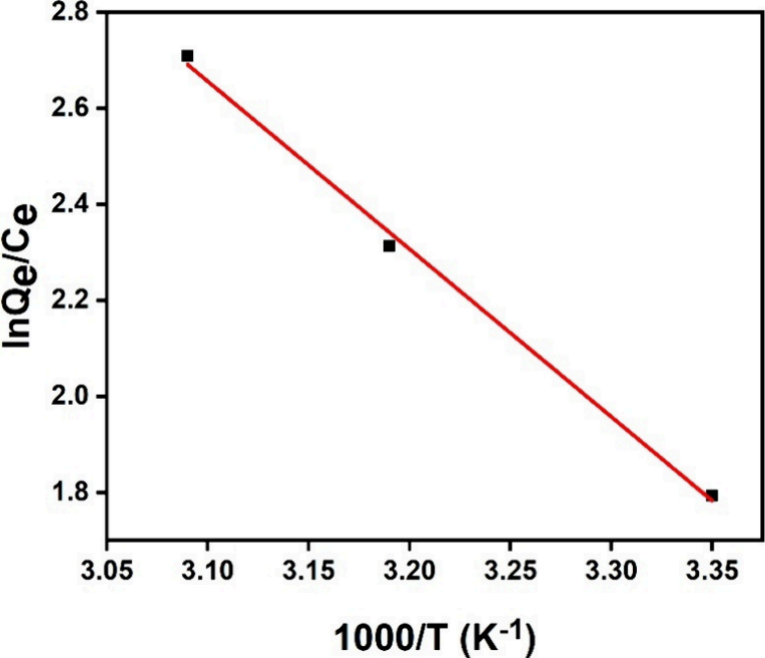
Thermodynamic study of the MO adsorption.

Another key parameter, Δ*H* ^0^, reflects the nature of the adsorption process. A positive Δ*H* ^0^ (32.14 kJ/mol) signifies an endothermic process^59^ and suggests weak interactions between the adsorbates and adsorbents.^60^ The endothermic nature of the process is attributed to the stronger interactions between the adsorbent and water molecules compared to those between the MOFs and methyl orange molecules.

The parameter Δ*S* ^0^, which indicates randomness, was also analyzed. A positive Δ*S* ^0^ reflects increased randomness during MO adsorption, as more water molecules are desorbed than MO molecules are adsorbed.^61^

These kinetic, isotherm, and thermodynamic analyses confirm the superior adsorption performance of the UiO-66-NH_2_/GO@PVA composite, further supported by comparative studies of its components (Figure 12). The composite exhibits the highest *Q*_m_ (188.63 mg/g), indicating significant synergistic effects among UiO-66-NH_2_, GO, and PVA. The binary hybrid UiO-66-NH_2_@GO also shows enhanced adsorption (159.72 mg/g), confirming the beneficial interaction between the MOF and graphene oxide. However, UiO-66-NH_2_ alone shows a lower *Q*_m_ (122.43 mg/g) and GO exhibits even lower performance (92.89 mg/g), likely due to fewer active sites or less interaction with the adsorbate. PVA displays the lowest adsorption capacity (80.89 mg/g), consistent with its limited intrinsic adsorption ability. These results clearly demonstrate that combining components into a ternary composite substantially improves the adsorption performance.

**Figure 12:**
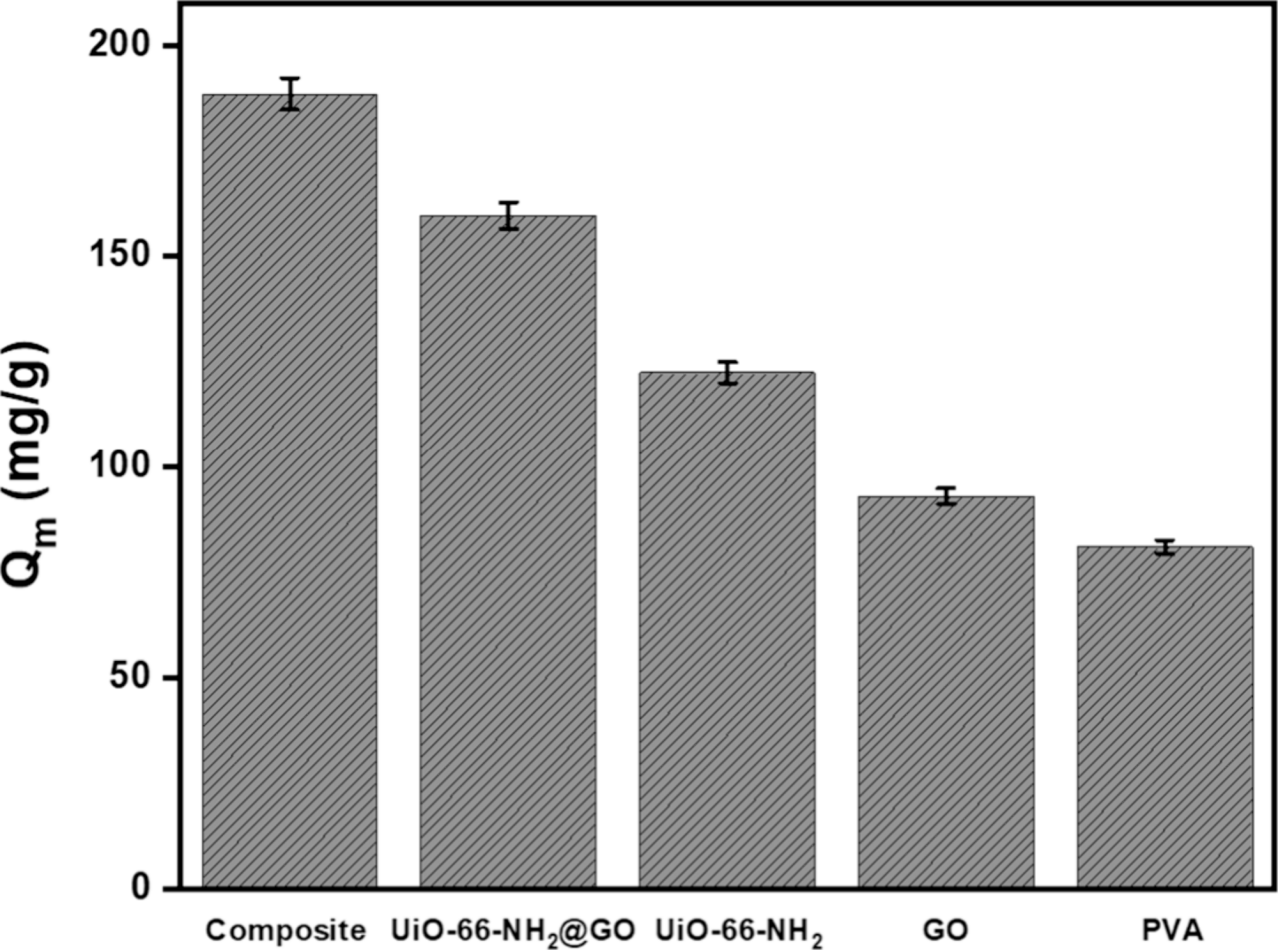
Comparative maximum adsorption capacities of UiO-66-NH_2_, GO, PVA, UiO-66-NH_2_@GO, and UiO-66-NH_2_/GO@PVA (pH 5, 100 mg/L MO, and 120 min of equilibrium).

### 3.4 Adsorption Mechanism

MO is an organic anionic dye in aqueous solution that is adsorbed onto UiO-66-NH_2_@GO@PVA via a heterogeneous mechanism, as supported by the Elovich kinetic model. The adsorption of MO onto UiO-66-NH_2_@GO@PVA involves a mixed mechanism, where physisorption occurs through the composite’s highly porous structure (2.7 nm pores, Figure 3b) and large surface area (1129 m^2^/g), contributing to spontaneous adsorption driven by van der Waals forces, as supported by Gibbs free energy changes (Δ*G* ^0^: −4.44 to −7.27 kJ/mol, Table 5). Additionally, because of the electrostatic attraction on the surface of the MOFs, MOFs can absorb MO molecules. The solution pH significantly influences the adsorption mechanism. At more acidic pH, the material surface is more positively charged, increasing electrostatic attraction with negatively charged MO molecules. Under slightly acidic conditions, the surface of MOFs becomes protonated.^62^ As the pH level rises, the adsorption ability of MO on the sample declines because of the enhanced negative charge on the surface of the MOFs, which repels MO anions in solutions. Additionally, the increasing OH^–^ concentration competes with MO anions for adsorption sites, further reducing adsorption efficiency. Therefore, a slightly acidic to neutral pH range is ideal for the maximal adsorption capability. In this study, a pH of 5 was selected.

The FTIR analysis of UiO-66-NH_2_@GO@PVA and MO before and after adsorption shows important interactions (Figure 14b). A large peak at 3374 cm^–1^ before adsorption is associated with O–H and N–H extending from PVA and NH_2_ groups. Its decrease in intensity following adsorption indicates that MO and the adsorbent have formed a hydrogen bond.^63^ Because of the C=C stretching in UiO-66-NH_2_, the signal at 1566 cm^–1^ changes somewhat, suggesting that MO’s sulfonate (−SO_3_^–^) groups and the amine (−NH_2_) sites are electrostatically interacting.^64^ Minor shifts in the peaks at 1254–1159 cm^–1^, which are linked to C–N stretching, validate chemical interactions. Furthermore, new peaks observed at 769–660 cm^–1^ indicate π–π stacking interactions between MO’s benzene rings and GO, corresponding to aromatic C–H bending.^65^ These results validate that physical adsorption, electrostatic attraction, π–π interactions, and hydrogen bonding control MO adsorption onto MOFs (Figure 13).

**Figure 13:**
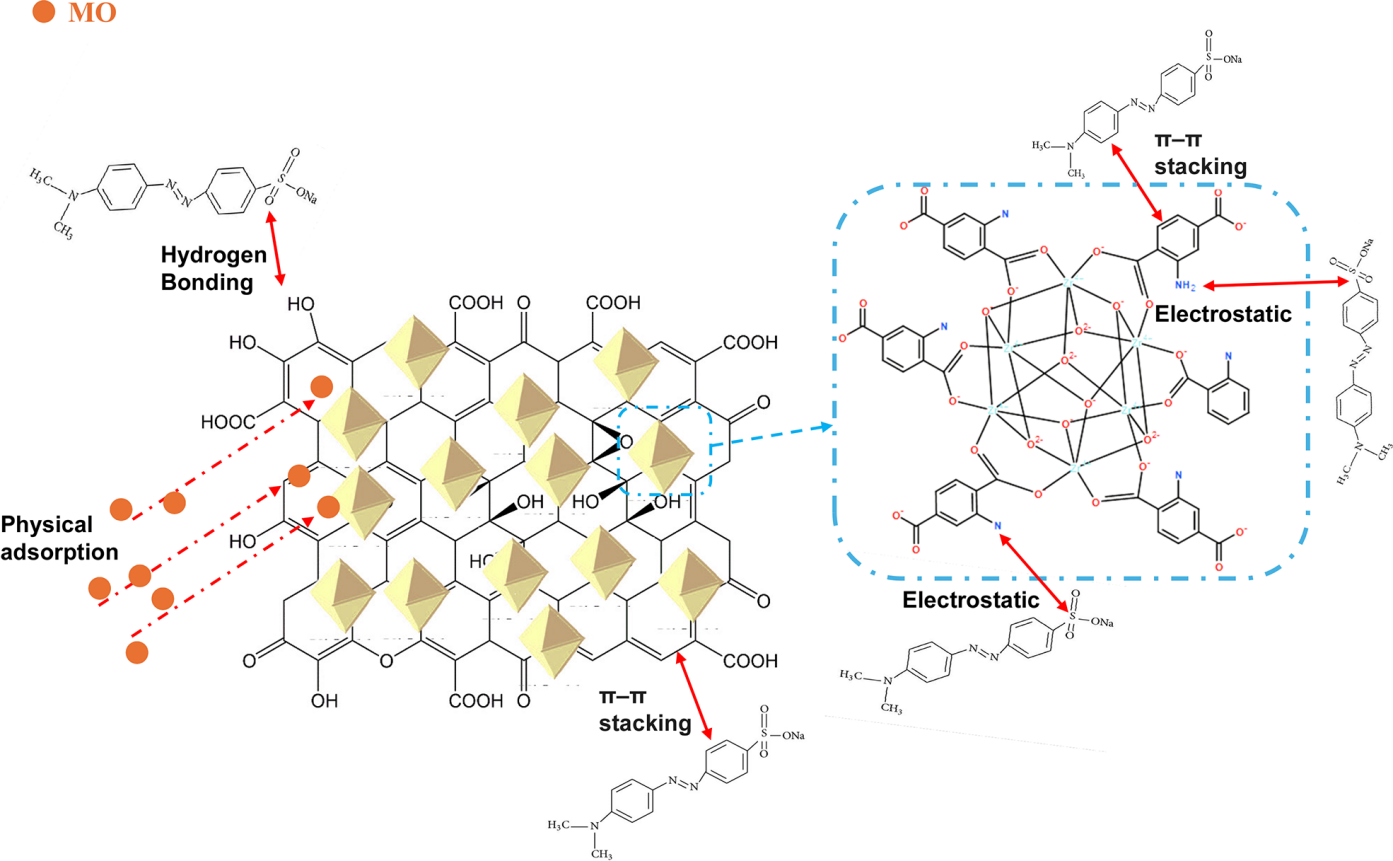
Adsorption mechanism of the MO onto UiO-66-NH_2_@GO@PVA.

### 3.5 Regeneration of Adsorbents

The practical use of adsorbents on an industrial scale depends on their reusability. It is also a practical way to treat wastewater in an economically and ecologically responsible manner. This work demonstrated the remarkable MO adsorption effectiveness of the MOFs composite matrix, which achieved 99.6% for the first time under 60 min. At the end of the third reuse, the adsorption effectiveness was still above 97%. Although it steadily declined after the fourth cycle, the MO adsorption capacity was still greater than 95% (Figure 14a). Regeneration was performed by washing the composite with 50 mL of ethanol (99.9%) at 25 °C for 2 h under stirring (120 rpm), followed by rinsing with 50 mL of deionized water and drying at 60 °C for 12 h. This process was standardized by maintaining consistent solvent volume, stirring speed, washing time, and drying conditions, with FTIR observations (Figure 14b) revealing characteristic peaks of MOFs after four cycles, highlighting the durability of the composite matrix over multiple cycles.^66,67^

**Figure 14:**
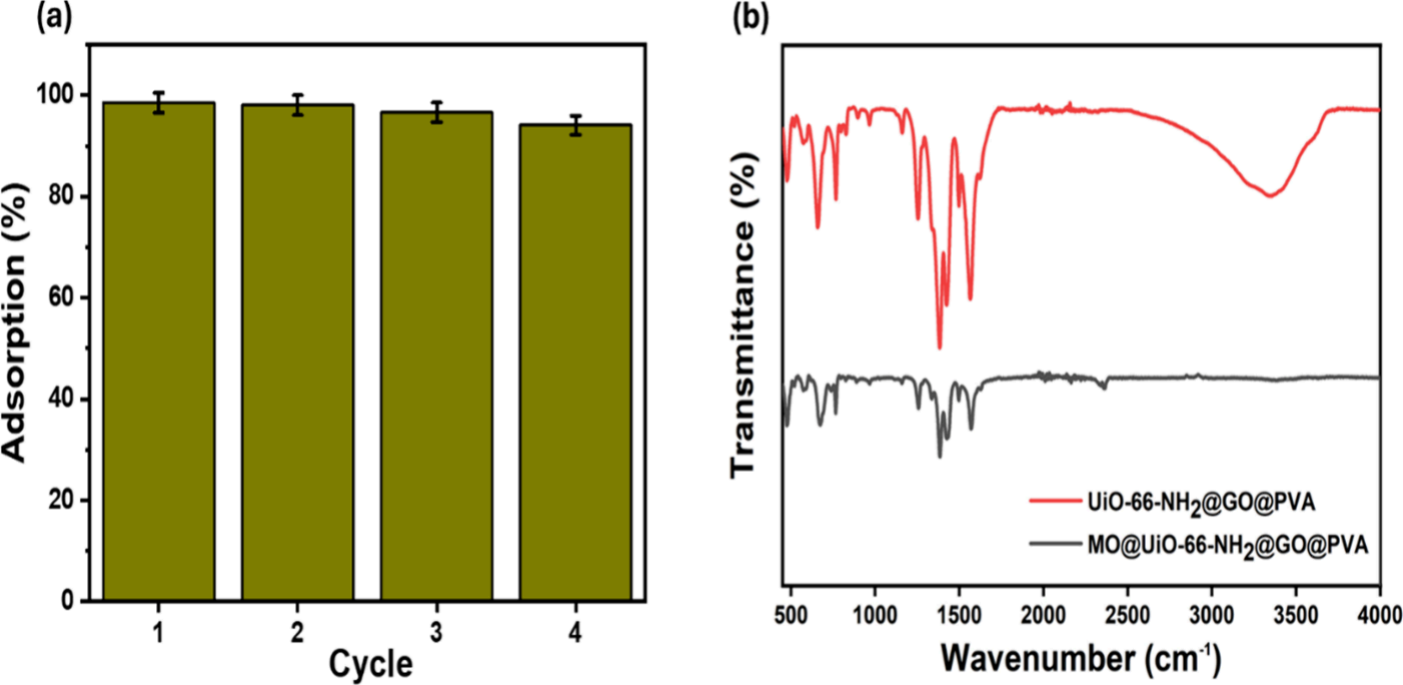
(a) Adsorbent regeneration cycles. (b) FTIR analysis after four adsorption cycles.

## 4 Conclusions

In this paper, the UiO-66-NH_2_/GO/PVA nanocomposite was successfully synthesized and applied for the elimination of methyl orange in aqueous solutions, achieving a maximum adsorption capacity of 188.63 mg/g and 99.6% efficiency at 100 mg/L and pH 5, as determined by the Langmuir isotherm model (*R* ^2^ = 0.9439). Kinetic studies, best fitted by the Elovich model (*R* ^2^ = 0.9999), confirmed a heterogeneous adsorption process with activated chemisorption, while thermodynamic analysis (Δ*G* ^0^: −4.44 to −7.27 kJ/mol, Δ*H* ^0^: 32.14 kJ/mol) indicated a moderately spontaneous, endothermic process, with physisorption driving the mechanism via porous structure and borderline chemisorption contributions from kinetic (pseudo-second-order, Elovich) and isotherm (Langmuir) models, suggesting a mixed mechanism. The composite retained over 95% adsorption efficiency after four regeneration cycles, demonstrating a consistent performance under repeated use. The novelty of UiO-66-NH_2_/GO@PVA lies in its ternary structure, where graphene oxide enhances dispersion and adsorption sites, and polyvinyl alcohol improves mechanical stability and porosity, as validated by the comparative analysis in Table 2. This composite outperforms other MOF-based materials, including Ni@ZIF-67 (151.74 mg/g, >90% efficiency after 5 cycles) and ZIF-8/0.5GO (82.78 mg/g), and surpasses the reusability of PAA–PVA/PW_12_@UiO-66 NFM (>92% efficiency after 5 cycles, capacity not mentioned). This positions UiO-66-NH_2_/GO@PVA as a significant advancement over these adsorbents, offering higher capacity and robust performance under repeated use.

These results suggest potential applicability in addressing dye pollution, a pressing global environmental challenge. The synergistic combination of UiO-66-NH_2_, graphene oxide, and poly­(vinyl alcohol) enhances the material’s adsorption capacity, mechanical stability, and porosity, setting it apart from conventional adsorbents. Control experiments with UiO-66-NH_2_ (122.43 mg/g), GO (92.89 mg/g), PVA (80.89 mg/g), and UiO-66-NH_2_@GO (159.72 mg/g) confirmed the ternary UiO-66-NH_2_/GO@PVA’s superior adsorption capacity (188.63 mg/g) due to synergy, validating its enhanced performance over individual compounds. Future research will focus on evaluating the selectivity and robustness of the proposed method under real or simulated wastewater conditions to further validate its practical applicability. This study lays a foundation for developing next-generation adsorbents by integrating polymer-MOF composites for sustainable removal of dye from industrial effluents.

## Data Availability

All the data supporting this article has been included in the research article. If the raw data is required, it will be made available on request.
